# Comparison of VMAT‐SABR treatment plans with flattening filter (FF) and flattening filter‐free (FFF) beam for localized prostate cancer

**DOI:** 10.1120/jacmp.v16i6.5728

**Published:** 2015-11-08

**Authors:** Jin‐Beom Chung, Jae‐Sung Kim, Keun‐Yong Eom, In‐Ah Kim, Sang‐Won Kang, Jeong‐Woo Lee, Jin‐Young Kim, Tae‐Suk Suh

**Affiliations:** ^1^ Department of Radiation Oncology Seoul National University Bundang Hospital Seongnam Korea; ^2^ Department of Biomedical Engineering The Catholic University of Korea Seoul Korea; ^3^ Department of Radiation Oncology Konkuk University Hospital Seoul Korea; ^4^ Department of Radiation Oncology Inje University Haeundae Paik Hospital Pusan Korea

**Keywords:** prostate cancer, stereotactic ablative radiotherapy, flattening filter‐free beam

## Abstract

The purpose of this study is to investigate the feasibility of using a flattening filter‐free (FFF) beam with an endorectal balloon for stereotactic ablative body radiotherapy (SABR) of clinically localized prostate cancer. We assessed plans of SABR with volumetric‐modulated arc therapy (VMAT) that used a flattening filter (FF) beam and an FFF beam and compared the verification results of dosimetric quality assurance for all pretreatment plans. A total of 20 patients with prostate cancer were enrolled in the study. SABR plans using VMAT with two full arcs were optimized in the Eclipse treatment planning system. All plans prescribed 42.7 Gy in 7 fractions of 6.1 Gy each. Four SABR plans were computed for each patient: two with FF beams and two with FFF beams of 6 and 10 MV. For all plans, the cumulative dose‐volume histograms (DVHs) for the target volumes and organs at risk (OARs) were recorded and compared. Pretreatment quality assurance (QA) was performed using the I'm*RT* MatriXX system and radiochromic EBT3 film to verify treatment delivery, and gamma analysis was used to quantify the agreement between calculations and measurements. In addition, total monitor units (MUs) and delivery time were investigated as technical parameters of delivery. All four plans achieved adequate dose conformity to the target volumes and had comparable dosimetric data. The DVHs of all four plans for each patient were very similar. All plans were highly conformal with CI<1.05 and CN>0.90, and the doses were homogeneous (HI = 0.08–0.15). Sparing for the bladder and rectum was slightly better with the 10 MV FF and FFF plans than with the 6 MV FF and FFF plans, but the difference was negligible. However, there was no significant difference in sparing for the other OARs. The mean agreement with the 3%/3% mm criterion was higher than 97% for verifying all plans. For the 2%/2% mm criterion, the corresponding agreement values were more than 90%, which showed that the plans were acceptable. The mean MUs and delivery time used were 1701±101 and 3.02±0.17 min for 6 MV FF, 1870±116 and 2.01±0.01 min for 6 MV FFF, 1471±86 and 2.68±0.14 min for 10 MV FF, and 1619±101 and 2.00±0.00 min for 10 MV FFF, respectively. In the current study, the dose distributions of the prostate SABR plans using 6 and 10 MV FFF beams were similar to those using 6 and 10 MV FF beams. However, this study confirmed that SABR treatment using an FFF beam had an advantage with respect to delivery time. In addition, all pretreatment plans were verified as acceptable and their results were comparable. Therefore, the results of this study suggest that the use of an FFF beam for prostate SABR is a feasible and efficient technique, if carefully applied.

PACS numbers: 87.55.D, 87.55.dk

## I. INTRODUCTION

Prostate cancer is the most common cancer in men, accounting for over one‐fifth of male cancer diagnoses, with the number of prostate cancer patients on the rise recently. Various radiotherapy techniques for treating prostate cancer have been considered effective, noninvasive treatment options, especially for elderly patients and those unfit for surgery.^1^, ^2^, ^3^

Stereotactic ablative body radiotherapy (SABR) with volumetric‐modulated arc therapy (VMAT) is an external beam radiation therapy method that delivers in a highly precise manner a high dose of radiation to an extracranial target within the body, in either a single dose or a small number of fractions. It is an attractive approach to dose escalation. The faster delivery of VMAT makes the treatment patient‐friendly and improves treatment accuracy because intrafractional motion is reduced. A previous study showed that VMAT improved delivery time and thus produced a highly conformal dose distribution and accurate dose delivery.^(4)^ In addition, VMAT was associated with more efficient monitor use, while maintaining optimized dose coverage and conformity to the planning target volume (PTV), when compared to intensity‐modulated radiotherapy (IMRT).

Recently, a new linear accelerator (linac) called TrueBeam (Varian Medical Systems, Palo Alto, CA) with flattening filter‐free (FFF) beams was introduced into clinical operation. There are two benefits to removing the flattening filter: 1) fast delivery time because of the high dose rates, which means the possibility of fast beam delivery in SABR treatment, and 2) reduction of the out‐of‐field dose as a result of reduced head scatter and leakage, which leads to reduced exposure of normal tissue to scattered doses outside the target field.^5^, ^6^, ^7^, ^8^, ^9^ A flattening filter (FF) beam is not required for VMAT because of the superposition of multiple‐intensity patterns. An FFF beam is frequently used when higher‐fraction doses are needed, especially in hypofractionated SABR of the lung, liver, and other sites.^10^, ^11^, ^12^, ^13^, ^14^, ^15^ The use of an FFF beam for SABR and three‐dimensional conformal radiotherapy (3D CRT) has been shown to be feasible.^(11)^ In a previous report,^(6)^ we presented our early experience with the use of FFF beams in the SABR treatment of lung cancer.

There are many reports on the use of VMAT in prostate cancer,^16^, ^17^, ^18^ but few regarding the use of FFF beams for prostate SABR with VMAT.^19^, ^20^, ^21^, ^22^, ^23^, ^24^ In this study, we tested the feasibility of the clinical use of FFF beams compared to FF beams in SABR treatment of patients with clinically localized prostate cancer. The cumulative dose‐volume histograms (DVHs) for the PTV and organs at risk (OARs) obtained with the use of FFF and FF beams were analyzed to evaluate the quality of the dosimetric plan. In addition, we assessed technical parameters, such as total monitor units (MUs) and delivery times, and evaluated pretreatment verification via the gamma agreement using two difference devices.

## II. MATERIALS AND METHODS

From 2013 to 2014, a total of 20 prostate cancer patients were enrolled in our SABR planning study, which was approved by the institutional review board (No. B‐1501/284‐107).

### A. Contouring and planning for SABR

A computed tomography (CT) (Brilliance CT Big Bore, Philips, Eindhoven, The Netherlands) simulation was performed on the patients, who were placed in a supine position on a flat bench and stabilized with Kneefix and Feetfix (CIVCO Medical Solutions, Coralville, IA). For the SABR simulation, the patients were asked to drink 300 ml of water 1 hr before simulation to ensure that the bladder was completely filled. An endorectal balloon (ERB) was inserted into the rectum and filled with 70 cc of air. After 1 min, the ERB catheter was placed at the premarked position and the inflated ERB was immobilized above the anal sphincter. A detailed description of the patient setup was given in our previous study.^16^, ^25^ The CT scans were acquired with 3 mm slice spacing.

The prostatic bed was delineated as the clinical target volume (CTV), and the PTV was defined as the CTV plus treatment margin of 7 mm posteriorly and 10 mm in all other directions, reflecting margins compatible with cone‐beam CT. The rectum, bladder, and femoral head were defined as the OARs. The rectum was defined as extending from the sigmoid flexure to the bottom of the ischium.

The Eclipse treatment planning system (v. 11.0.34, Varian Medical Systems) was used for the planning of VMAT with SABR. Two full arcs of the rotating system deliver a highly conformal dose to the PTV while maximally sparing adjacent OARs. To simplify and standardize VMAT treatment planning for prostate SABR, class solutions have been devised.^(26)^ The prescription dose was 42.7 Gy given in 7 fractions. This delivers a higher biologically effective dose (BED) to the prostate, but at a dose that is equivalent to late‐responding dose compared to 78 Gy in 39 fractions, which is the standard prostate fractionation.

The maximum available dose rate was used for each beam — 600 MU/min for FF beams, 400 for 6 MV FFF, and 2,400 for 10 MV FFF. All dose distributions were calculated using Acuros XB (AXB, Varian, version 11) and 2.5 mm isotropic dose grid.

For all cases, the objective of the planning was to cover at least 95% of the PTV with 95% of the prescribed dose ($V_{95\%}\geq 95\%$). The optimal constraints for the OARs in prostate SABR remain unknown. In this study, we used modified constraints for the OARs derived from those reported by Murray et al.^(26)^ that were suitable for our clinic. The constraints for the OARs for this planning study are listed in Table 1.

**Table 1:** Dose volume constraints adopted for planning study

*Volume*	*Constraints*
Rectum	$V_{42.7\;\text{Gy}}<5\%$
	$V_{38.4\;\text{Gy}}<15\%$
	$V_{32.0\;\text{Gy}}<35\%$
	$V_{28.0\;\text{Gy}}<45\%$
	$V_{24.8\;\text{Gy}}<70\%$
	$V_{20.0\;\text{Gy}}<80\%$
Bladder	$V_{42.7\;\text{Gy}}<10\%$
	$V_{34.7\;\text{Gy}}<25\%$
	$V_{29.9\;\text{Gy}}<50\%$
Femoral heads	$V_{29.9\;\text{Gy}}<50\%$
	$D_{\max}<29.9\;\text{Gy}$

$D_{\max}=\text{the}\;\text{maximum}\;\text{dose}$; ${\text{Vx}}_{\text{Gy}}=$ volume receiving xGy dose.

### B. Dosimetric and technical data evaluation

We compared the cumulative dose‐volume histograms (DVHs) and technical parameters for all cases. In addition, we measured the mean, maximum, and minimum doses for the PTV. To represent the target coverage, $V_{95\%}$ for PTV (i.e., the volume of PTV receiving more than 95% of the prescribed dose) and $V_{100\%}$ for CTV were evaluated. Several conformity indices were analyzed to confirm the homogeneity of the plans. The homogeneity index (HI) of the PTV (as defined by the International Commission on Radiation Units and Measurements report 83^(27)^) was defined as $D_{2\%}-D_{98\%})/D_{50\%}$, where $D_{2\%}$ is the maximum dose received by 2% of the PTV, $D_{98\%}$ is the minimum dose received by 98% of the PTV, and $D_{50\%}$ is the dose received by 50% of the PTV. A low HI means the plan is more homogeneous because $D_{2\%}$ and $D_{98\%}$ are surrogates for maximum dose and minimum dose in the PTV, respectively.^(28)^ The conformity index (CI) was defined as the ratio of the patient volume irradiated by 95% of prescribed dose to the PTV. The ideal conformation is defined as CI=1, and when CI>1, healthy tissues have been irradiated.^(29)^ The conformation number (CN) takes into consideration the irradiation of healthy tissue. It is the product of two fractions, ${\text{TV}}_{\text{RI}}/\text{TV}$ and ${\text{TV}}_{\text{RI}}/V_{\text{RI}}$, where TV is the PTV, ${\text{TV}}_{\text{RI}}$ is the volume of the PTV covered by the reference isodose line, and $V_{\text{RI}}$ is the volume enclosed by the reference isodose line. ${\text{TV}}_{\text{RI}}/\text{TV}$ is the quality of the target coverage and ${\text{TV}}_{\text{RI}}/V_{\text{RI}}$ is the volume of healthy tissue irradiated with the reference isodose or more.^(30)^ We used 95% isodose as the reference isodose line. For the OARs, we investigated the near‐to‐maximum dose ($D_{2\%}$) and the mean dose. In addition, we performed a detailed analysis of the rectum and bladder volumes that received at least $95\%(V_{95\%})$, $80\%(V_{80\%})$, $50\%(V_{50\%})$, and $20\%(V_{20\%})$ of the prescribed dose; these values represent very high, high, intermediate, and low doses, respectively.

All four plans underwent pretreatment verification using the I'm*RT* MatriXX 2D array (IBA Dosimetry GmbH, Schwarzenbruck, Germany) with a MultiCube phantom to measure the coronal plane and radiochromic EBT3 film Gafchromic (International Specialty Products, Wayne, NJ) with a homemade cylindrical acrylic phantom to measure the axial plane. Film dosimetry was used to improve the spatial resolution and to compare the axial dose distribution.^31^, ^32^ To quantify the differences between the calculated and measured dose distributions, we used gamma analysis to determine the agreement scores using a 3 mm distance to agreement (DTA) and a 3% relative dose difference (3%/3% mm) for the criteria. The point percentage when gamma is <1 is a measure of the agreement between the measurements and the calculations. To study the effect of different criteria on the passing rate, we used the criteria 2%/2% mm, which are used clinically for IMRT and VMAT quality assurance. In addition, total MUs and delivery time were recorded for each plan.

## III. RESULTS

### A. Dosimetric data of target volumes and OARs

**Figure 1:**
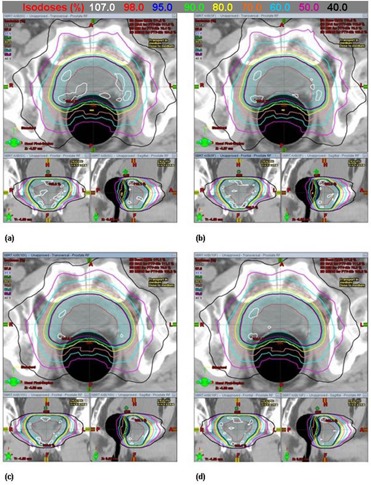
Example of the dose distributions from prostate SABR plans using (a) 6 MV FF, (b) 6 MV FFF, (c) 10 MV FF, and (d) 10 MV FFF beams for the same patient.

**Figure 2:**
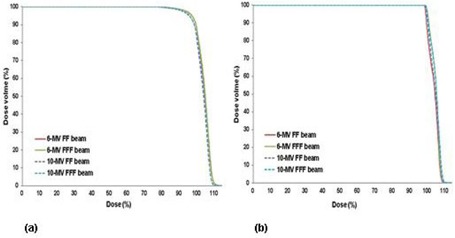
The average dose‐volume histograms of (a) PTV and (b) CTV in SABR plans using 6 MV FF, 6 MV FFF, 10 MV FF, and 10 MV FFF beams for all patients.

**Figure 3:**
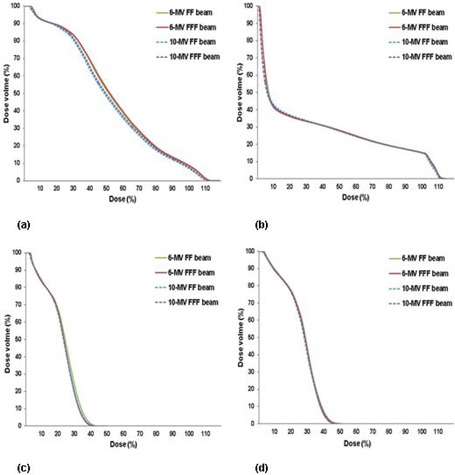
The average dose‐volume histograms of (a) rectum, (b) bladder, and (c) left and (d) right femoral heads in SABR plans using 6 MV FF, 6 MV FFF, 10 MV FF, and 10 MV FFF beams for all patients.

Figure 1 shows an example of the dose distributions achieved with 6 MV FF, 6 MV FFF, 10 MV FF, and 10 MV FFF beams for the same patient. Figures 2 and 3 show the average DVHs for CTV and PTV, and all OARs in the SABR plans with the four different beams. The DVHs for all four plans for each patient were very similar. There were small differences in the dose distributions and corresponding DVHs among the four plans of the same patient. Tables 2 and 3 summarize the dosimetric results from DVH analysis of target volumes and various OARs (i.e., bladder, rectum, and left and right femoral head).

There were no significant differences in CTV and PTV coverage among the four plans, with the exception of the 10‐MV FFF beam plan, where there was a significant increase in $V_{100\%}$ of CTV from the 6 MV FF to the 10 MV FFF plan: 96.66% vs. 98.65%. All plans were highly conformal with CI<1.05 and CN>0.90, and the doses were homogeneous (HI=0.08–0.15). The difference in the maximum dose of PTV for plans with the same energy (6 MV FF vs. 6 MV FFF and 10 MV FF vs. 10 MV FFF) was, on average, 0.9 Gy.

With respect to the dose to the OARs, sparing for the bladder and the rectum was slightly better with the 10 MV FF and FFF beam plans than with the 6 MV FF and FFF beam plans; however, this difference was negligible except for rectum volume that received the moderate dose ($V_{50\%}$). Table 3 and Fig. 3 show that the moderate dose volume to the rectum was 3.2% less on average for the 10 MV FF and FFF beams than for the 6 MV FF and FFF beams. There was no significant difference in dose volume between the left and right femoral heads for all plans generated by different beams.

**Table 2:** Summary of dosimetric results for PTV and CTV in prostate SABR plans using 6 MV FF, 6 MV FFF, 10 MV FF, and 10 MV FFF beams for all patients

	Mean±SD (min−max)			
	*6 MV FF*	*6 MV FFF*	*10 MV FF*	*10 MV FFF*
*CTV Coverage*				
$D_{50\%}\;(\text{Gy})$	44.4±0.6(43.2−45.6)	44.5±0.6(43.1−45.6)	44.2±0.6(43.0−45.3)	44.7±0.6(43.3−45.4)
$D_{2\%}\;(\text{Gy})$	46.4±0.4(45.7−46.9)	46.5±0.4(46.0−47.1)	46.2±0.6(45.4−47.3)	46.3±0.4(45.8−47.1)
$D_{98\%}\;(\text{Gy})$	42.8±0.5(42.3−44.1)	42.8±0.5(42.2−44.2)	42.8±0.6(42.1−44.1)	43.2±0.6(42.1−44.4)
$V_{100\%}(\%)$	96.7±.3(85.2−100.0)	96.8±3.7(86.6−100.0)	97.0±3.4(89.9−100.0)	98.7±2.4(93.0−100.0.0)
*PTV Coverage*				
$D_{\text{mean}}$	44.4±0.2(43.8−44.7)	44.4±0.2(43.7−44.8)	44.0±0.3(43.4−44.4)	44.2±0.2(43.8−44.5)
$D_{\text{max}}$	49.6±1.0(48.4−51.8)	49.2±0.9(47.6−50.9)	48.7±0.9(47.2−50.7)	48.3±0.7(47.2−49.7)
$D_{\min}$	32.1±0.8(30.3−33.2)	32.3±0.9(30.5−33.4)	32.1±1.1(29.9−34.3)	32.1±1.1(30.0−35.3)
$D_{50\%}\;(\text{Gy})$	44.6±0.4(43.8−45.2)	44.6±0.4(43.7−45.3)	44.3±0.4(43.4−44.9)	44.5±0.4(43.6−45.0)
$D_{2\%}\;(\text{Gy})$	47.0±0.4(46.3−47.8)	46.9±0.3(46.4−47.5)	46.6±0.4(46.0−47.4)	46.5±0.3(46.1−47.1)
$D_{98\%}\;(\text{Gy})$	39.8±0.8(38.3−41.1)	39.9±0.7(37.5−41.1)	39.1±1.0(37.3−41.0)	39.4±0.9(37.5−42.0)
$D_{95\%}(\%)$	41.7±0.3(41.1−42.3)	41.6±0.2(38.5−42.1)	41.1±0.4(40.4−42.0)	41.1±0.3(40.7−41.9)
Conformity Index	1.05±0.03(1.00−1.11)	1.05±0.03(1.00−1.12)	1.04±0.03(1.00−1.10)	1.03±0.02(1.00−1.08)
Conformation Number	0.90±0.02(0.84−0.92)	0.91±0.02(0.83−0.93)	0.90±0.02(0.82−0.96)	0.91±0.02(0.84−0.96)
Homogeneity Index	0.11±0.02(0.08−0.15)	0.11±0.02(0.08−0.15)	0.12±0.03(0.08−0.16)	0.10±0.02(0.08−0.12)

${\text{Dx}}_{\%=}$ dose received by at least x% of the volume; ${\text{Vx}}_{\%=}$ volume receiving at least x% of prescription dose; $D_{\text{mean}}$ = the mean dose; $D_{\text{min}}$ = the minimum dose; $D_{\text{max}}$ = the maximum dose.

**Table 3:** Summary of dosimetric results for organs at risk in prostate SABR plans using 6 MV FF, 6 MV FFF, 10 MV FF, and 10 MV FFF beams for all patients

	Mean±SD(min−max)			
	*6 MV FF*	*6 MV FFF*	*10 MV FF*	*10 MV FFF*
*Organs At Risk*				
Rectum $D_{\text{mean}}$ (Gy)	23.1±2.4(18.1−26.4)	23.0±2.3(17.8−25.9)	22.4±2.3(17.3−25.3)	22.5±2.4(17.2−25.2)
Rectum $D_{2\%}$ (Gy)	44.4±0.5(42.9−45.2)	44.7±0.3(43.8−45.2)	43.7±0.4(42.7−44.5)	44.1±0.5(43.6−44.6)
Rectum $V_{95\%}$ (%)	8.9±1.7(4.8−11.5)	9.1±1.7(5.0−11.6)	8.4±1.9(4.4−13.4)	7.9±2.0(3.9−13.1)
Rectum $V_{80\%}$ (%)	17.9±3.7(10.1−24.2)	17.8±3.4(10.2−23.4)	17.0±3.3(10.2−22.9)	17.4±3.5(9.6−22.9)
Rectum $V_{50\%}$ (%)	54.2±11.3(34.7−77.7)	54.4±11.2(34.4−78.5)	51.2±10.4(36.1−77.0)	51.6±10.7(31.9−75.6)
Rectum $V_{20\%}$ (%)	88.5±6.4(70.2−95.6)	88.4±6.7(68.7−96.4)	88.2±6.5(69.8−95.3)	88.2±6.7(68.7−96.8)
Bladder $D_{\text{mean}}$ (Gy)	17.5±6.1(7.9−33.1)	17.46±6.13(7.8−33.1)	17.3±6.1(7.7−33.0)	17.3±6.2(7.6−33.2)
Bladder $D_{2\%}$ (Gy)	45.7±0.6(44.8−47.4)	45.7±0.5(44.9−47.1)	45.2±0.5(44.3−46.2)	45.6±0.5(44.8−46.4)
Bladder $V_{95\%}$ (%)	20.2±12.0(4.9−58.4)	20.2±12.1(4.8−58.4)	19.8±11.8(4.7−57.5)	19.9±12.0(4.8−58.1)
Bladder $V_{80\%}$ (%)	23.7±13.1(6.8−65.2)	23.6±13.1(6.7−65.2)	23.5±13.0(6.8−65.0)	23.5±13.2(6.8−65.3)
Bladder $V_{50\%}$ (%)	30.2±15.5(14.0−77.5)	30.3±15.6(13.9−77.7)	29.5±15.3(13.6−77.5)	29.6±15.4(13.5−77.8)
Bladder $V_{20\%}$ (%)	37.2±17.1(26.8−81.6)	38.8±16.9(26.2−82.3)	37.7±17.3(26.8−83.1)	37.6±17.4(26.5−85.9)
Left femoral head $D_{\text{mean}}$ (Gy)	12.6±2.3(8.8−14.6)	12.5±2.4(8.6−14.6)	12.5±2.4(8.7−14.9)	12.4±2.4(8.8−14.9)
Left femoral head $D_{2\%}$ (Gy)	18.1±2.5(13.5−24.5)	18.0±2.6(13.4−24.1)	18.2±2.6(14.0−25.0)	18.0±2.6(13.4−24.8)
Right femoral head $D_{\text{mean}}$ (Gy)	12.8±2.1(9.5−16.9)	12.8±2.1(9.4−16.7)	12.8±2.2(9.3−17.2)	12.8±2.2(9.3−15.5)
Right femoral head $D_{2\%}$ (Gy)	18.7±2.3(14.4−23.9)	18.5±2.4(14.3−23.6)	18.8±2.5(14.5−24.6)	18.7±2.5(14.3−24.6)

${\text{Dx}}_{\%=}$ dose received by at least x% of the volume; ${\text{Vx}}_{\%=}$ volume receiving at least x% of prescription dose; $D_{\text{mean}}$ = the mean dose; $D_{\text{min}}$ = the minimum dose; $D_{\text{max}}$ = the maximum dose.

### B. Verification of pretreatment plans

**Figure 4:**
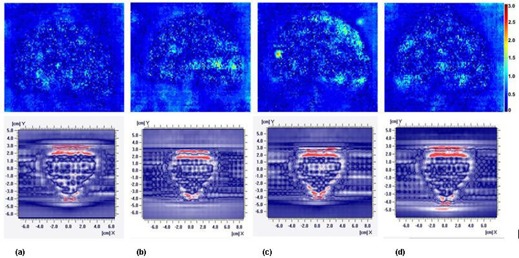
The spatial distribution of gamma analyses with 3%/3% mm criteria for film (upper row) and I'MatriXX system (lower row) of case 1. From left to right, the results are for (a) 6 MV FF, (b) 6 MV FFF, (c) 10 MV FF, and (d) 10 MV FFF beams, respectively.

Figure 4 shows the spatial distribution of the gamma analyses with the 3%/3% mm criteria for the I'm*RT* MatriXX system and the film irradiated by prostate SABR for case 1. Table 4 summarizes and compares the results of the gamma evaluations between the measurements and the calculations of the planar dose. For pretreatment verification with film on the axial plane for all plans, the mean gamma agreement scores for the 3%/3% mm criteria were 99.27%, 98.25%, 97.42%, and 98.60% for 6 MV FF, 6 MV FFF, 10 MV FF, and 10 MV FFF beam plans, respectively. The mean gamma agreement scores for the I'm*RT* MatriXX system on the coronal plane were 97.43%, 98.12%, 97.34%, and 97.37%. These data show that the difference between the measured dose and the calculated dose is acceptable and the results are comparable for all plans. The mean passing rates for the 3%/3% mm criteria were higher than 97% on the coronal plane and the axial plane, and there were no statistical differences for the four different beam plans. For the 2%/2% mm criteria, the corresponding agreement values were greater than 90%, which showed that the plans were acceptable.

**Table 4:** The results of the gamma evaluation in all pretreatment plans using 6 MV FF, 6 MV FFF, 10 MV FF, and 10 MV FFF beams

		*Percentage of Pixels with Gamma* <1			
*Verification*	*Criteria*	6 MV FF (Mean±SD)	6 MV FFF (Mean±SD)	10 MV FF (Mean±SD)	10 MV FFF (Mean±SD)
Film	3%/3% mm	99.1±1.1	98.3±1.1	97.0±1.1	98.6±1.0
(Axial plane)	2%/2% mm	93.3±2.1	90.9±2.5	90.3±2.4	93.4±2.3
Matrixx	3%/3% mm	97.4±1.1	98.1±1.0	97.3±1.0	97.4±1.0
(Coronal plane)	2%/2% mm	91.0±2.5	92.9±2.0	90.8±2.2	91.4±2.0

FFF=flattening filter‐free; FF=flattening filter; SD=standard deviation.

### C. Technical data of treatment plans

Table 5 presents the technical data for the prostate SABR plans with the four different beams. The mean MUs and the delivery time were 1701±101 and 3.02±0.17 min for the 6 MV FF beam, 1870±116 and 2.01±0.01 min for the 6 MV FFF beam, 1471±86 and 2.68±0.14 min for the 10 MV FF beam, and 1619±101 and 2.00±0.00 min for the 10 MV FFF beam, respectively. The FFF beam plans required more MUs than the FF beam plans. The ratio of the MUs for 6 MV FF, 10 MV FF, and 10 MV FFF beams to that for the 6 MV FFF beam were 0.910, 0.787, and 0.866, respectively, and the ratios of treatment delivery time were 2.19, 1.94, and 1.00 respectively. The delivery time was remarkably shorter with the 6 MV and 10 MV FFF beams.

**Table 5:** The results of technical parameters in prostate SABR plans using 6 MV FF, 6 MV FFF, 10 MV FF, and 10 MV FFF beams for all patients

*Beams*	6 MV FF (Mean±SD)	6 MV FFF (Mean±SD)	10 MV FF (Mean±SD)	6 MV FFF (Mean±SD)
MU	1701±101	1870±116	1471±86	1619±101
Delivery time (min)	3.02±0.17	2.01±0.01	2.68±0.14	2.00±0.00

FFF = flattening filter‐free; FF = flattening filter; SD = standard deviation; MU = monitor unit.

## IV. DISCUSSION

The use of SABR to irradiate primary or metastatic tumors in several anatomical sites is becoming the standard treatment. For instance, the use of SABR for the treatment of early‐stage lung cancer has been reported with promising clinical results.^4^, ^33^ Bignardi et al.^(34)^ and Scorsetti et al.^(35)^ reported a good toxicity profile and clinical results from their study of the technical feasibility, local control rate, and acute toxicity of SABR with VMAT used on patients with primary or secondary abdominal tumors.

A previous study reported that large radiation fraction sizes are radiobiologically favorable over lower fraction sizes in prostate cancer.^(36)^ Recent evidence also suggests that prostate tumors have a low α/β ratio (estimated as ~ 1.5–2 in the prostate vs. 3 in the rectum), making it theoretically more sensitive to dose‐per‐fraction treatments.^3^, ^37^ For a very low α/β ratio, a linear quadratic model suggests that SABR delivered with ultrahypofractionation is an attractive approach to improve the therapeutic ratio (i.e., the ratio of the maximally tolerated dose to the minimally curative or effective dose) of radiotherapy for prostate cancer. Currently, the data on VMAT‐SABR treatment with FFF beams for prostate cancer are limited. In this study, we compared the dosimetric effects, technical parameters, and pretreatment verification for a VMAT‐SABR prostate plan with two beam types (FF and FFF beams) and two beam energies (6 and 10 MV).

The DVHs of the target volumes (PTV and CTV) with FF and FFF beams of both energies were very similar. In 19 cases in this study, the target coverage satisfied the criteria for the planning objective (i.e., V98%>98% of CTV and V95%>95% of PTV). In only one case the criteria were not met because a large portion of the PTV included the air‐filled endorectal balloon that was used to reduce the intrafractional motion and improve sparing of the rectal wall. Kang et al.^(38)^ reported that target coverage is difficult for a prostate IMRT plan that has an area with air in the PTV. In addition to the presence of air in the PTV, our patient's plan required the highest MUs.

The effect of the dose difference on the OARs was negligible; however, the maximum mean dose difference was within 9.07% of $D_{50\%}$ for the rectum with the 6 MV FF beam compared to that of the 10 MV FF beam (see Fig. 3(a)). The magnitude of this difference for OARs sparing depended on the beam energy. Rectum and bladder doses were reduced by <3% when 10 MV beam was used instead of the 6 MV FF and FFF beams. However, the doses were similar for the left and right femoral heads. The overall dose differences for the OARs were not significant for all four plans.

More than 90% of the gamma analysis criteria for meeting the passing rate were defined as acceptable. The verification of the FF beam and the FFF beam plans showed that they yielded acceptable results (Fig. 4 and Table 4). The percentage of pixels that failed the 3%/3% mm criteria within the region of interest, averaged over all measurements and plans, was within 3% for all pretreatment plans; for the 2%/2% mm criteria, it was within 10%. The dose distributions of the 6 MV FF and 10 MV FFF beam SABR plans showed fairly good agreement with the measured dose distributions, with small differences. In general, our pretreatment verification results for the FF and FFF beams were comparable and in agreement with previously published results.^(39)^

The FFF beam plan required more MUs than the FF beam plan with the same energy. The MUs ratio of FF beam to FFF beam was 0.910 for 6 MV and 0.909 for 10 MV. This is in line with the result from a previous study^(15)^ on a lung SABR plan, where the MUs value of the FFF beam was up to 10% greater than that of the FF beam, and the delivery time was reduced by a factor of 2.5. The significant benefit of using an FFF beam is the reduction of treatment delivery time. In another study^(16)^ in which only the prostate bed was considered, the treatment delivery time was about 3 min, whereas in the current study, the treatment delivery times for 6 MV and 10 MV FFF beams were reduced up to 2 min compared to 3 min in conventional 6 MV FF beam. Reducing delivery time reduces intrafractional motion and decreases the patient's discomfort. However, the mean delivery time was similar for 6 MV and 10 MV FFF plan using two 360° arcs because the delivery duration is limited by the gantry rotation speed and leaf speed, not the dose rate.

One limitation of this study is that there are no definitive clinical data on short‐ and long‐term outcomes. This study focused mainly on the feasibility and efficiency of using the FFF beam in SABR treatment for prostate cancer. Therefore, follow‐up studies are needed to confirm the clinical outcome and toxicity of prostate SABR treatment using FF and FFF beams.

## V. CONCLUSIONS

From a dosimetric perspective, prostate SABR plans using 6 and 10 MV FFF beams were similar to those generated with 6 and 10 MV FF beams. However, this study demonstrated that the plans using 6 and 10 MV FFF beams offer a clear benefit with respect to treatment delivery time when compared to the plans using 6 and 10 MV FF beams. Verification of the pretreatment plans showed that all FF and FFF plans had acceptable and dosimetrically comparable results. Therefore, the results of this study suggest that the use of the FFF beam is feasible and efficient in SABR treatment for prostate cancer, if carefully applied, confirming safety of the clinical use for the acute and late toxicity relative to high dose intensity of FFF beam. Furthermore, use of a 10 MV FFF beam for prostate SABR provides slightly better sparing of OARs than a 6 MV FFF beam.

## ACKNOWLEDGMENTS

This study was supported by a Korea Heavy Ion Medical Accelerator project grant by the Korean government (Ministry of Education, Science and Technology, MEST) (Grant no. 2014M2C3A1029534) and the Radiation Technology R&D program through the National Research Foundation of Korea funded by the Ministry of Science, ICT and Future Planning (Grant no. 2013M2A2A7043498).
